# Physico-Chemical and Biological Features of Fluorine-Substituted Hydroxyapatite Suspensions

**DOI:** 10.3390/ma17143404

**Published:** 2024-07-10

**Authors:** Carmen Steluta Ciobanu, Daniela Predoi, Simona Liliana Iconaru, Mihai Valentin Predoi, Krzysztof Rokosz, Steinar Raaen, Catalin Constantin Negrila, Nicolas Buton, Liliana Ghegoiu, Monica Luminita Badea

**Affiliations:** 1 National Institute of Materials Physics, Atomistilor Street, No. 405A, 077125 Magurele, Romania; simonaiconaru@gmail.com (S.L.I.); catalin.negrila@infim.ro (C.C.N.); ghegoiuliliana@gmail.com (L.G.); 2 Department of Mechanics, University Politehnica of Bucharest, BN 002, 313 Splaiul Independentei, Sector 6, 060042 Bucharest, Romania; predoi@gmail.com; 3 Faculty of Electronics and Computer Science, Koszalin University of Technology, Sniadeckich 2, PL 75-453 Koszalin, Poland; rokosz@tu.koszalin.pl; 4 Department of Physics, Norwegian University of Science and Technology (NTNU), Realfagbygget E3-124 Høgskoleringen 5, NO 7491 Trondheim, Norway; steinar.raaen@ntnu.no; 5 HORIBA Jobin Yvon S.A.S., 6–18, Rue du Canal, 91165 Longjumeau CEDEX, France; nicolas.buton@horiba.com; 6 Faculty of Horticulture, University of Agronomic Sciences and Veterinary Medicine, 59 Marasti Blvd., 011464 Bucharest, Romania; badea.artemisia@gmail.com

**Keywords:** fluorine substituted hydroxyapatite, suspensions, ultrasound measurements, stability, biocompatibility, antimicrobial properties

## Abstract

Infections related to orthopedic/stomatology surgery are widely recognized as a significant health concern. Therefore, the development of new materials with superior biological properties and good stability could represent a valuable alternative to the classical treatments. In this paper, the fluorine-substituted hydroxyapatite (FHAp) suspension, with the chemical formula Ca_10_(PO_4_)_6_(OH)_2−2x_F_2x_ (where x = 0.05), was prepared using a modified coprecipitation technique. Stability studies were conducted by zeta potential and ultrasound measurements for the first time. The X-ray diffraction (XRD) patterns of FHAp powders displayed a hexagonal structure akin to that of pure hydroxyapatite (HAp). The XPS general spectrum revealed peaks corresponding to the constituent elements of fluorine-substituted hydroxyapatite such as calcium, phosphorus, oxygen, and fluorine. The purity of the obtained FHAp samples was confirmed by energy-dispersive X-ray spectroscopy (EDS) studies. The FHAp morphology was evaluated by scanning electron microscopy (SEM) measurements. Fourier-transform infrared spectroscopy (FTIR) studies were performed in order to study the vibrational properties of the FHAp samples. The FHAp suspensions were tested for antibacterial activity against reference strains such as *Staphylococcus aureus* 25923 ATCC, *Escherichia coli* ATCC 25922, and *Candida albicans* ATCC 10231. Additionally, the biocompatibility of the FHAp suspensions was assessed using human fetal osteoblastic cells (hFOB 1.19 cell line). The results of our biological tests suggest that FHAp suspensions are promising candidates for the future development of new biocompatible and antimicrobial agents for use in the biomedical field.

## 1. Introduction

Nowadays, hydroxyapatite (HAp) has attracted the attention of the scientific community, especially as a crucial substitute/coating material for biomedical applications (e.g., orthopedics and dentistry applications, etc.) [1,2]. The use of hydroxyapatite in various biomedical applications is attributed to its excellent bioactivity, biocompatibility, and osteoconductivity, as well as its chemical and biological similarity to the mineral component of bone tissue [1,2]. According to previous studies, the enhancement of the HAp features can be achieved through the incorporation of various dopants in their structure [3,4]. HAp possesses a structure that permits the replacement of ions at the Ca^2+^ and OH^−^ sites with various cations and anions [5,6]. Methods such as wet precipitation or the solid-state method have been employed to develop new HAp-based biomaterials doped with Ag^+^, Cu^2+^, Mg^2+^, Zn^2+^, and F^−^, as documented in prior research [7,8,9,10,11,12]. Fluoride (F^−^), is a trace element found in natural bone, blood, and dental enamel, and it is known to promote and enhance new bone growth and formation [13]. On the other hand, fluoride plays a key role in the prevention of dental caries by strengthening the resilience of tooth surface minerals against acidic dissolution under the oral cavity in low pH conditions [14,15]. Various synthesis methods have been employed to develop fluorine-substituted hydroxyapatite (with various F ion concentration), each yielding to different morphologies and degrees of crystallinity, as follows: wet chemical methods, hydrothermal methods, the multiple emulsion technique, sol–gel, wet precipitation, microwave synthesis, the dry solid-state method, the electrode deposition technique, the modified wet chemical process and mechanochemical techniques [15]. Among these synthesis methods, the one based on the wet chemical precipitation technique has gained popularity due to its simplicity, cost-effectiveness, and ease of application in industrial manufacturing [16].

*Staphylococci* cause around 80% of orthopedic implant-associated infections, and *Staphylococcus aureus* (*S. aureus*) is responsible for one-third of them [17]. Also, *Candida* species can infect orthopedic implants [17]. Furthermore, both *S. aureus* and *Candida albicans* have the ability to form biofilms, which is a key step in the development of implant infections [17]. On the other hand, *Escherichia coli* (*E. coli*) is a leading cause of Gram-negative bacterial infections of orthopedic implants [18]. It is well known that infections associated with orthopedic implants pose a significant health issue.

A potential solution to this problem may be repented by the incorporation of antimicrobial substances into hydroxyapatite, which may prevent the growth and development of microbes on the implant surface [17,18]. The study conducted by Nasker, P. and coworkers [19] was focused on the development of hydroxyapatite nano-powders with various fluorine concentrations using hydrothermal processing and by analyzing their structure and effects on cell viability (using mouse osteoblast cell line (MC3T3-E1)) and bacterial growth (against Gram-negative (*Escherichia coli*) and Gram-positive (*Staphylococcus aureus*) microbial strains). Their findings underlined that the studied powders were non-toxic and that those with 50% or higher fluorine substitution impeded the proliferation of osteoblast cells [19]. Additionally, fluorine-substituted HAp exhibited moderate antibacterial properties [19].

In the research paper reported by Stanić, V. and colleagues [20], the results of fluorine-substituted hydroxyapatite obtained by the neutralization method were presented [20]. The results of antimicrobial tests showed that the efficiency of the fluorapatite materials against the *Streptococcus mutans* microbial strain depends on the fluoride concentration [20]. More than that, Tredwin, C.J. et al. [21] reported the fabrication of hydroxyapatite and fluor-hydroxyapatite (with various fluoride ion concentrations) the sol–gel method, along with in vitro biological assay outcomes on the human osteosarcoma (HOS) cell line [21]. Their results highlighted that a high fluoride concentration within the HAp structure leads to enhanced biocompatibility [21]. Another study conducted by S. Shanmugam and coworkers [22] revealed the superior antibacterial efficiency of fluorapatite against Gram-negative (*E. coli*), Gram-positive (*S. aureus*) and fungus (*C. albicans*) strains [22].

In this context, the novelty of this study presents the development of a fluorine-substituted hydroxyapatite (FHAp, Ca_10_(PO_4_)_6_(OH)_2−2x_F_2x_, x = 0.05) suspension by an adapted coprecipitation method and its complex characterization from both a physico-chemical and biological point of view. In this manuscript are reported, for the first time, the results of ultrasound studies conducted on FHAp suspensions that provide valuable information about their stability. Other techniques such as X-ray diffraction (XRD), Fourier transform infrared spectroscopy (FTIR), X-ray photoelectron spectroscopy (XPS), scanning electron microscopy (SEM), dynamic light scattering (DLS) and zeta potential (ZP) were employed in the FHAp sample characterization. The in vitro biological properties of FHAp suspensions were also evaluated.

## 2. Materials and Methods

### 2.1. Materials

Calcium nitrate (Ca(NO_3_)_2_·4H_2_O), diammonium hydrogen phosphate ((NH_4_)_2_HPO_4_), ammonium fluoride (NH_4_F), an ammonium hydroxide (NH_4_OH) 30% solution and double-distilled water were used in this study. The calcium nitrate, diammonium hydrogen phosphate and ammonium hydroxide were purchased from Sigma-Aldrich, St. Louis, MO, USA. The ammonium fluoride was purchased from Merck Romania S.R.L., Bucharest, Romania.

#### Synthesis of Fluorine Substituted Hydroxyapatite (FHAp)

The suspension of FHAp (Ca_10_(PO_4_)_6_(OH)_2−2x_F_2x_, x = 0.05) was obtained by an adapted co-precipitation method [11,12]. To obtain the suspension, a 0.5 M calcium nitrate solution was prepared. The aqueous solutions of diammonium hydrogen phosphate and ammonium fluoride were added drop by drop to the calcium nitrate solution under continuous stirring at 100 °C. The Ca/P ratio was 1.67. The pH during the synthesis was maintained at 10. The pH was kept constant by adding ammonium hydroxide (NH_4_OH) 30% solution. At the end of the drip, the resulting solution was stirred for 12 h at 100 °C. At the end, the formed precipitate was washed several times by centrifugation. After the last wash, the resulting precipitate was redispersed in 100 mL of double-distilled water under continuous stirring for 12 h. The resulting suspension was analyzed from a physico-chemical and biological point of view.

### 2.2. Characterization Techniques

#### 2.2.1. Ultrasound, Dynamic Light Scattering (DLS) and Zeta (ζ) Potential Studies

**Figure 1:**
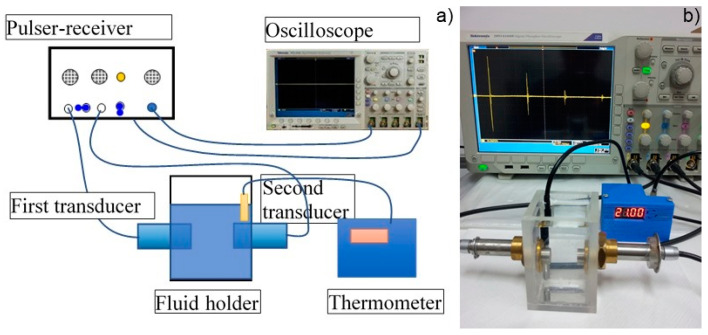
Experimental setup used for ultrasound measurements of FHAp suspension: schematics (**a**) and image (**b**) [23].

The stability of the FHAp suspension was evaluated by non-destructive ultrasound (US) studies. For these experiments, double-distilled water was used as reference. The protocol and instrument used for the US studies were described in our previous study [23]. The experimental setup used for the ultrasound measurements of the FHAp suspension is presented in Figure 1 [23]. Therefore, the spectral amplitude as a function of frequency for the FHAp suspension relative to the reference fluid (double-distilled water), in addition to attenuation as a function of frequency, were employed to assess the stability of the FHAp suspension.

The dynamic light scattering (DLS) and Zeta (ζ) potential studies were performed with the aid of a SZ-100 Nanoparticle Analyzer (Horiba-SAS France, Longjumeau, France) at 25 ± 1 °C [23]. For this purpose, the FHAp suspension was diluted in water 10 times prior to the measurements. The reported values represent the mean value of 3 determinations.

#### 2.2.2. X-ray Diffraction

The X-ray diffraction (XRD) patterns were registered using a Bruker D8 Advance diffractometer with CuKα radiation (λ = 1.5418 Å) (Bruker, Karlsruhe, Germany) equipped with a LynxEye™ 1D high-efficiency one-dimensional linear detector. Data regarding the FHAp powders were acquired in the 2θ range of 20–70° with a step size of 0.02° and a time of 5 s per step.

#### 2.2.3. Fourier Transform Infrared Spectroscopy

The Fourier-Transform Infrared spectroscopy (FTIR) spectra for the obtained sample were acquired at ambient temperature using a Perkin Elmer Spectrum BX II spectrometer (Waltham, MA, USA). The FTIR spectrometer was operated in ATR mode. The experimental data were recorded in a wavelength range between 3800 and 450 cm^−1^.

#### 2.2.4. X-ray Photoelectron Spectroscopy

The X-ray photoelectron spectroscopy (XPS) investigations were conducted using a SES 2002 instrument (Scienta Omicron, Taunusstein, Germany). The XPS measurements utilized a monochromatic Al K (alpha) X-ray source with an energy of 1486.6 eV. The scan analyses and the experiments respected protocols established in the previous studies [24,25]. The CasaXPS 2.3.14 software was used [26] for data analysis. All binding energy (BE) values presented in this study were charge-corrected to C1s at 284.8 eV.

#### 2.2.5. Scanning Electron Microscopy

Scanning electron microscopy (SEM) and energy-dispersive X-ray spectroscopy (EDS) were used to study the morphology and chemical composition of the FHAp suspension. For this purpose, a Hitachi S4500 microscope (Hitachi, Tokyo, Japan) equipped with an energy-dispersive X-ray spectroscopy system for detection and chemical analysis was employed.

### 2.3. In Vitro Biological Assays

#### 2.3.1. Antimicrobial Activity

The antibacterial activity of the FHAp suspensions was tested against the following reference strains: *Staphylococcus aureus* 25923 ATCC, *Escherichia coli* ATCC 25922, and *Candida albicans* ATCC 10231. The antimicrobial properties of the FHAp suspensions were determined using microbial suspensions of 1.5 × 10^8^ CFU/mL corresponding to a 0.5 McFarland density obtained from 15 to 18 h bacterial cultures developed on solid media [25,27]. A control was used using the non-inoculated culture media (sample blank). The absorbances were measured at 620 nm, and the microbial viability was represented as a percentage compared to the value obtained for the control sample.

(1) $$\mathrm{Microbial}\;\mathrm{cell}\;\mathrm{viability}=\frac{\;A\;sample-A\;sample\;blank}{A\;strain\;control-A\;blank}\;\times \;100$$

#### 2.3.2. Hemolysis

The hemocompatibility of the FHAp suspensions was evaluated by studying the hemolytic activity. The hemolysis assays were performed using sheep red blood cells (RBCs) using a modified procedure previously described by Das et al. [28]. For this purpose, a 5% RBC suspension was loaded into a tube and incubated at 37 °C for 30 min. Both water and a NaCl solution were added to the RBC suspensions to obtain the positive and negative controls, respectively. The FHAp suspensions at different concentrations were mixed with the RBC suspensions, and all the groups were incubated at 37 °C for 4 h. The hemolysis% of the FHAp suspensions was determined with the aid of the following equation:

(2) $$\mathrm{Hemolysis}\;(\%)=\frac{OD\;sample-OD\;negative\;control}{OD\;positive\;control-OD\;negative\;control}\;\times \;100$$

The hemolysis % assays were performed in triplicate and the results were depicted as mean ± SD.

Human fetal osteoblastic cells (hFOB 1.19 cell line) were grown in Ham’s F12: Dulbecco Modified Eagle’s Medium (1:1) without phenol red, with 2.5 mM of L-glutamine and 0.3 mg/mL of G418, and with 10% fetal bovine serum (Gibco, Waltham, MA, USA) at 37 °C in a humidified atmosphere with 5% CO_2_. The cells were seeded at a cell density of 3 × 10^4^ cells/ cm^2^ on the tissue culture plastic surface, which served as a control, or on the top of the tested samples, which were previously sterilized under UV light. After 24 h of incubation in standard conditions, biocompatibility tests were performed.

#### 2.3.3. MTT Assay

The biocompatibility of the FHAp suspensions was studied with the aid of Human fetal osteoblastic cells (hFOB 1.19 cell line). For this purpose, the cells were grown in Dulbecco Modified Eagle’s Medium enhanced with L-glutamine and with 10% fetal bovine serum (Gibco, USA) at 37 °C in a humidified atmosphere with 5% CO_2_. The cells were seeded at a cell density of 3 × 10^4^ cells/cm^2^ on the tissue culture plastic surface, which served as a control. The cellular viability was determined using the 3-(4,5-dimethylthiazol-2-yl)-2,5-diphenyltetrazolium bromide (MTT; Sigma-Aldrich, USA) assay. The cellular viability was evaluated at three different time intervals (24, 48 and 72 h). After each incubation period, the medium was removed and the cells were incubated with 1 mg/mL of MTT for 4 h at 37 °C. The absorbance was measured at 595 nm using a microplate reader (Flex Station 3, Molecular Devices, San Jose, CA, USA) and the cell viability was quantified from the absorbance values.

## 3. Results

**Figure 2:**
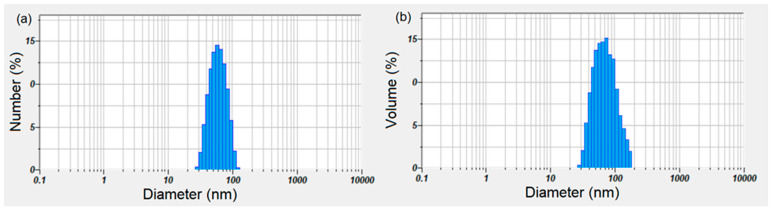
Dynamic light scattering (DLS) measurements of the size dispersion of the FHAp, showing data weighted by the number (**a**) and volume (**b**) of particles, respectively.

The size of the FHAp particles in suspension (hydrodynamic diameter, D_H_), as well as the stability of these suspensions, were analyzed by classical techniques such as dynamic light scattering (DLS) and zeta potential (ZP). For the FHAp suspensions, stability studies using ultrasonic measurements were also performed to confirm the stability of these suspensions. The Dynamic Light Scattering (DLS) technique and zeta potential are widely used in biochemistry, biotechnology and pharmaceutical development [29,30]. The DLS technique offers the ability to quickly and easily measure the distribution and size of nanoparticles. The disadvantage of this technique is that the analyzed particle suspensions must be diluted. However, by using the DLS technique, we can obtain information about the quality of the sample before using other expensive and time-consuming bio-physical methods. Since errors may appear in the analysis process as a result of the dilution of the suspension obtained following the synthesis process, in this study we used ultrasonic measurements. Ultrasonic measurements were performed on the concentrated suspensions obtained after dilutions. When the DLS data were weighted by numbers, the hydrodynamic diameter (DH) of the FHAp particles in suspension was 65.3 nm (Figure 2a). When the DLS data were weighted by volume, the D_H_ of the FHAp particles in suspension was 78.69 nm (Figure 2b). The measured zeta potential (ZP) value of the FHAp suspension was −27.58 mV, which indicates moderate stability. The measured value for the zeta potential shows that the FHAp suspension does not have good stability.

In order to obtain more accurate information on the stability of the FHAp suspension, ultrasound measurements were performed on undiluted suspensions. First, 100 mL of the FHAp suspension was poured into a special transparent cubical container. Two coaxial ultrasonic transducers were immersed in the suspension and were distanced by 16 mm, facing each other. The transducers’ axis was at 29 mm from the flat bottom of the container box. The FHAp suspension was continuously stirred for 15 min at 750 rot/min in order to obtain a good homogeneity of the solid particles. Immediately after stopping the magnetic stirring machine, the acquisition of the 2000 ultrasonic signals began, recorded every 5 s from the oscilloscope. Each recorded signal was an average of 32 signals on the oscilloscope, reducing the experimental noise.

**Figure 3:**
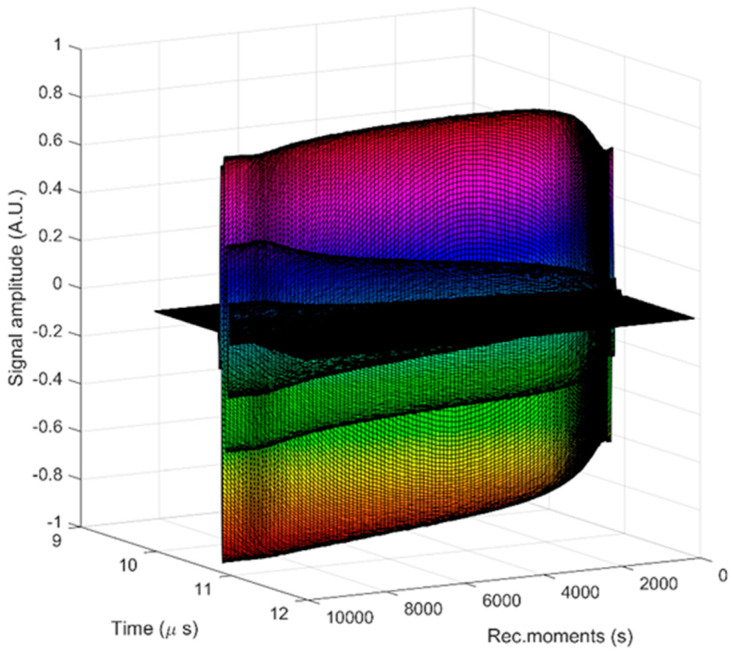
Time evolution of the recorded signals, from left to right over 10,000 s.

A superposition of the recorded signals is shown in Figure 3. From right to left, all 2000 signals covering 10,000 s of process evolution are plotted as water flow. The rapid initial evolution of amplitudes was followed by a progressive and slow variation in the overall amplitudes.

**Figure 4:**
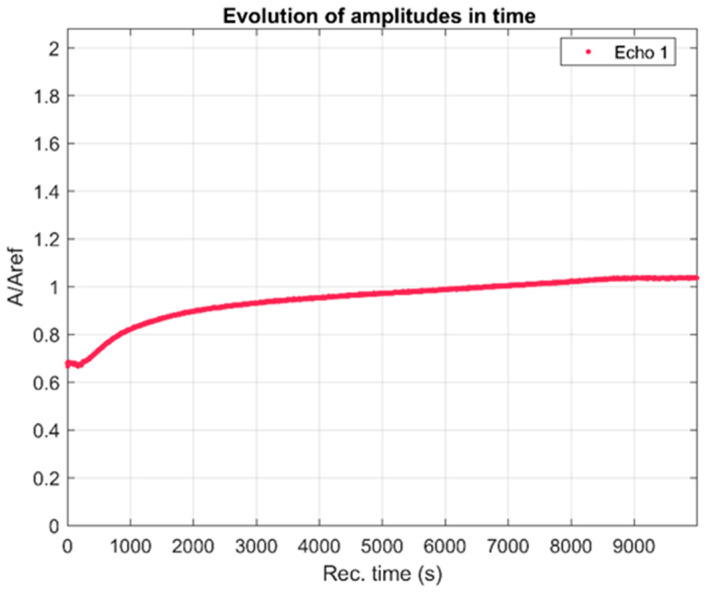
Recorded signal amplitudes during the experiment.

**Figure 5:**
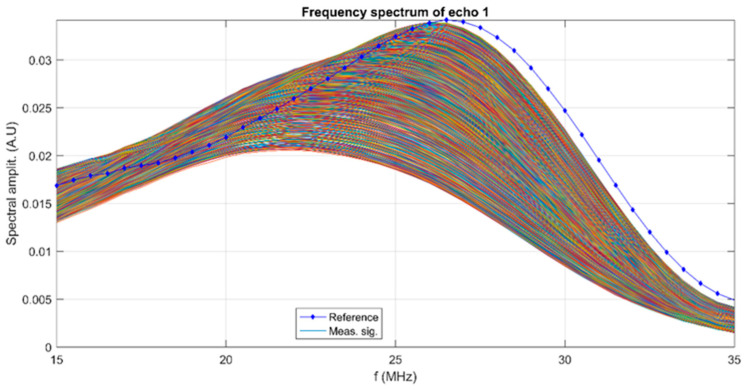
Spectral amplitudes of all 2000 signals.

This complex evolution is detailed in Figure 4. An initial small decrease is followed by a nonlinear increase in the measured signal amplitudes. The temporal change in properties manifests as a variation in the frequency spectra of all recorded FHAp sample signals. These spectra are shown in Figure 5 and, for comparison, the spectrum of the reference liquid is also plotted (double distilled water, in dotted blue line).

**Figure 6:**
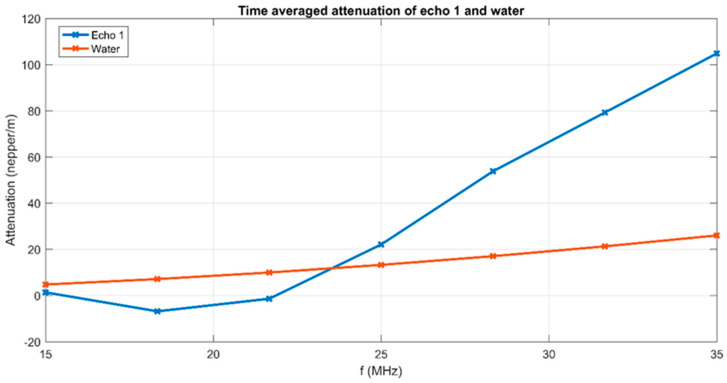
Time-averaged attenuation for the investigated frequency range.

The evolution of signals in time is represented by the wide distribution of the 2000 spectral curves, which differ significantly from the spectrum of the reference liquid, which has a peak at 26.2 MHz. The spectra evolve from ones with lower peak amplitudes of 0.021 at 22 MHz to amplitudes close to those of the reference liquid, at 26 MHz. This important evolution during the experiment indicates a continuous variation in the suspension concentration, which diminishes in time. The ultrasonic signals are also attenuated by the suspension. The time-averaged attenuation plot is shown in Figure 6. Compared against the standard attenuation in the reference liquid (red dotted line), the attenuation is much larger for the FHAp sample in the higher frequency ranges, reaching 103 nepper/m at 35 MHz. In the frequency range of 15–23.5 MHz, the attenuation is lower than the attenuation in the reference liquid.

**Figure 7:**
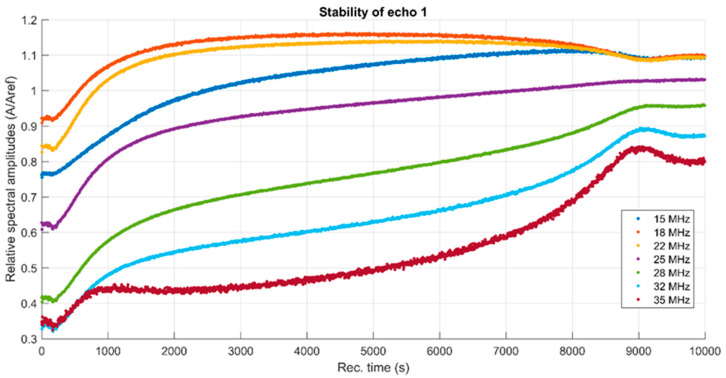
Relative spectral amplitudes vs. time.

Another FHAp suspension characteristic is the spectral stability, representing the amplitude of the frequency component of each spectrum, as a function of time (Figure 7).

Some remarkable features can be associated with this sample. During the first 100 s, all spectral amplitudes decrease, a fact that can be attributed to the settlement of the larger particles in suspension. Then, the amplitudes begin to rise in a more or less pronounced manner. For example, the 35 MHz spectral component is no longer following the curve at 32 MHz, but keeping a relatively constant amplitude up to 3000 s, followed by a pronounced increase. A specific feature is the tendency to concentrate the amplitudes after 9000 s of evolution, followed by a tendency to spread again, a phenomenon associated with the passage of the sedimentation plane in front of the transducer’s axis. The relative spectral amplitudes larger than the unit (value for the reference liquid) indicate that the suspension has not sedimented even after 10,000 s.

The FHAp suspension is not very stable; the stability parameter $S=\frac{\bar{dA}}{Adt}=0.00016\;s^{-1}$, in which A is the signal amplitude, with the bar above indicating averaging. This value confirms the continuous slow variation in properties during the test. The results obtained from ultrasound measurements regarding the stability of FHAp suspensions were in agreement with those obtained from zeta potential measurements. The information regarding the stability of suspensions is very important for biological materials because stability directly influences their efficiency [31,32].

**Figure 8:**
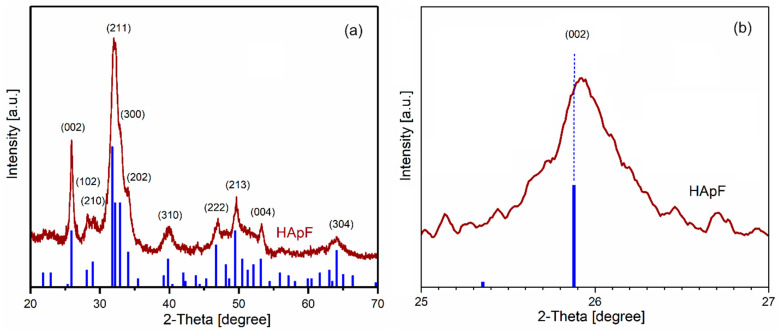
The peaks associated with the JCPDS # 09–0432 card (blue lines) and X-ray diffraction pattern of FHAp powder (**a**); relative shift in (002) in FHAp (**b**).

The phase composition, lattice parameters, and crystallite size of the FHAp powder obtained by an adapted coprecipitation method were determined by comparing the XRD pattern (Figure 8a) with the standard JCPDS cards for hydroxyapatite (HAp, 9-0432). The X-ray diffraction pattern of the FHAp powder revealed the peaks (002), (210), (211), (300), (202), (310), (222), (213), and (004), showing the formation of hexagonal pure HAp (space group P63/m). The lattice constants were determined through least-squares refinements using the well-defined positions of the most intense reflections, namely (002), (211), (300), (202) and (310). Based on the information provided, it appears that the diffraction maxima moved slightly to the right. This fact was very well observed in the case of the (002) plane when the 2θ value changed from 25.879° (according to JCPDS #09-0432) to 25.942° (Figure 8b). The diffraction spectrum did not exhibit peaks corresponding to other phases.

This behavior could be due to the incorporation of fluorine in the hydroxyapatite structure, as shown in previous reports in the literature [33,34,35]. Moreover, S. Kannan et al. [34] argued that the replacement of OH- ions placed in disordered positions with negatively charged F^−^ ions leads to a change in the network parameters. The calculated lattice constants of the FHAp sample were a = b = 0.9358 nm and c = 0.6861 nm. The calculated crystallite size of FHAp was 19.55 nm. The X-ray diffraction (XRD) patterns of the FHAp powders exhibited a hexagonal structure similar to pure hydroxyapatite (in agreement with the JCPDS cards for hydroxyapatite, 9-0432). This resemblance suggests that FHAp retains the fundamental crystallographic arrangement characteristic of HAp. The obtained result is in full agreement with previous studies in which it was shown that when analyzing apatite powders with substituted elements using X-ray diffraction, the resulting diffraction patterns can resemble those of hexagonal hydroxyapatite (HAp), even if there are significant differences in the element concentrations [34,36,37,38,39].

**Figure 9:**
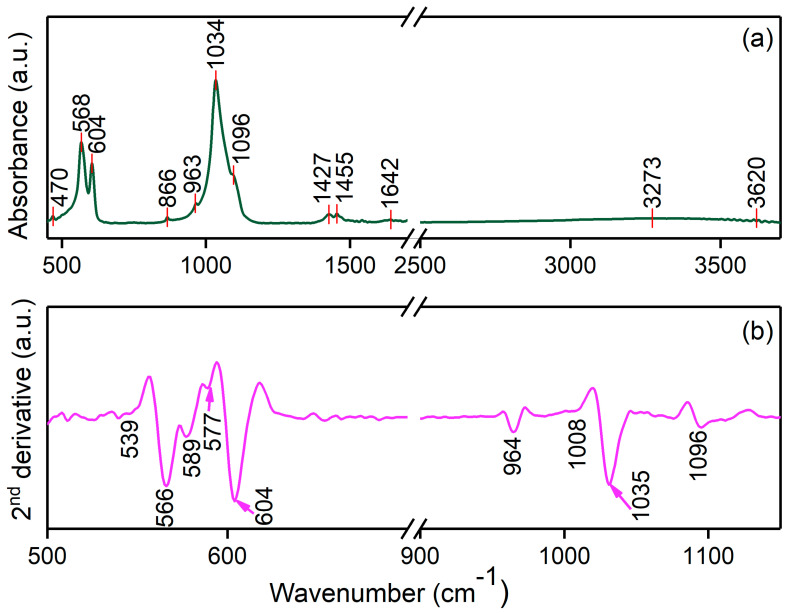
FTIR (**a**) and second derivative (**b**) spectra obtained for FHAp.

In Figure 9a,b, both the FTIR (absorbance) and 2nd derivative spectra of the FHAp sample are presented. The results of the FTIR studies reveal the presence of the HAp in the analyzed sample without highlighting the formation of new phases following the substitution with F ions. Thus, in the FTIR spectra (Figure 9a), the maxima that belong to the PO_4_^3−^ groups are observed at approximately 470 cm^−1^ (O–P–O symmetric bending, ν_2_), 568 cm^−1^ and 604 cm^−1^ (O–P–O antisymmetric bending, ν_4_), 963 cm^−1^ (P–O symmetric stretching, ν_1_), and around 1034 cm^−1^/1096 cm^−1^ (P–O antisymmetric stretching, ν_3_) [25,40,41,42,43]. Additionally, the maxima that could be attributed to carbonate groups (ν_3_ antisymmetric, CO_3_^2−^) are evident at around 1455 and 1427 cm^−1^ [25,40,41,42,43]. Furthermore, the maxima observed at 1642 cm^−1^ could be attributed to the H–O–H bands of the water lattice [44]. The maxima observed in the 3200–3600 cm^−1^ spectral domain are usually associated with the antisymmetric and symmetric vibrations of water and the OH group within the HAp lattice [45].

The second derivative (SD) studies allowed the differentiation of peaks within the absorption FTIR spectrum that are normally too tightly spaced to be separately identified [46]. We performed these SD studies in the next spectral domains: 500 cm^−1^–700 cm^−1^ and 900 cm^−1^–1150 cm^−1^. It could be observed that there was no split in the weak maxima centered at 964 cm^−1^ (that belongs to the ν_1_ vibration of phosphate groups in the HAp structure) [46]. In the ν_4_(PO_4_^3−^) region, sive sub-bands centered at 539 cm^−1^, 655 cm^−1^, 589 cm^−1^, 577 cm^−1^ and 604 cm^−1^ were detected [46]. No other intense sub-bands were detected in this spectral domain. The second derivative spectra obtained for the 900 cm^−1^–1150 cm^−1^ spectral domain was dominated by the sub-band centered at 1035 cm^−1^. In the same spectral domain, the sub-bands centered at 1008 cm^−1^ and 1096 cm^−1^ were observed. All these sub-bands could be attributed to the ν_3_ (PO_4_^3−^) from the hydroxyapatite lattice [46]. The lack of additional significant sub-bands suggests that the studied FHAp samples are pure. These FTIR and SD findings are in good agreement with the ones previously reported by Leung, Y. et al. [46].

**Figure 10:**
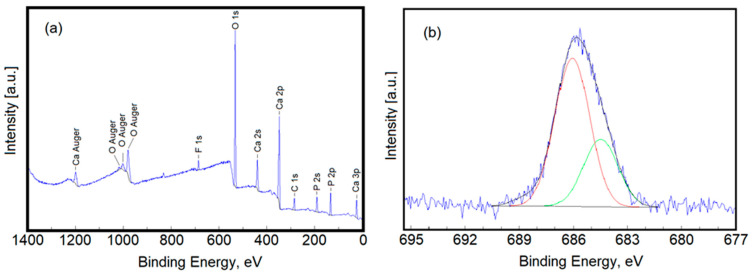
XPS general spectrum (**a**) and high-resolution spectra of F1s (**b**).

The XPS general spectrum of FHAp was collected and is shown in Figure 10a. The XPS general spectrum of FHAp shows peaks corresponding to the constituent elements: calcium (Ca), phosphorus (P), oxygen (O), and fluorine (F). The F1s × ray photoelectron spectroscopy of the samples and its fitting results are also examined in Figure 10b. The narrow XPS scan of F1s revealed two peaks located at 684.2 eV and 686.6 eV, respectively. In agreement with previous studies [47,48], the peak located at 684.2 eV is the imprint of F^−^ in the FHAp structure and certifies the fact that fluoride ions were successfully incorporated into the HAp structure. The peak observed at 686.6 eV is due to the reaction between Ca^2+^ and F^−^ ions, in agreement with previous studies [49,50].

**Figure 11:**
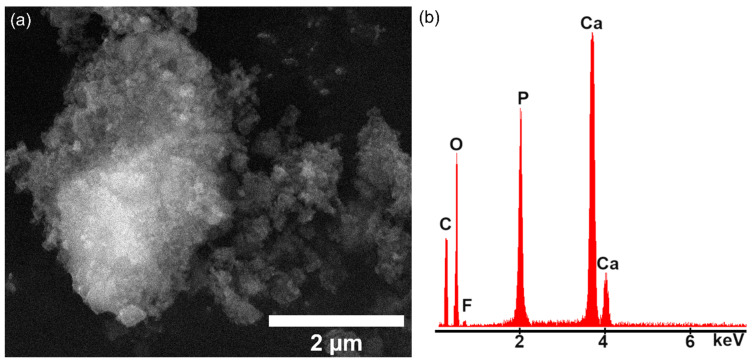
SEM micrographs (**a**) and EDS spectra (**b**) of FHAp (Ca_10_(PO_4_)_6_(OH)_2−2x_F_2x_, x = 0.05).

Information about the FHAp samples’ morphology was obtained by SEM. For these studies, a drop of FHAp suspension was placed on the carbon tape and dried prior to SEM examination. The results of the scanning electron microscopy studies and EDS studies are presented in Figure 11. The SEM micrographs of FHAp reveal that the particles were obtained at a nanometric scale and had a pronounced tendency to form agglomerates.

Information about the chemical composition of the FHAp suspension was obtained via EDS studies and their results are shown in Figure 11b. The main line observed in the EDS spectra belongs to the calcium (Ca K), phosphate (P K), oxygen (O K) and fluorine (F K). All these chemical elements are the main constituents of the FHAp structure. No other noticeable lines could be observed in Figure 11b, which suggests the FHAp’s purity. These results are consistent with the ones obtained in XPS and XRD studies.

**Figure 12:**
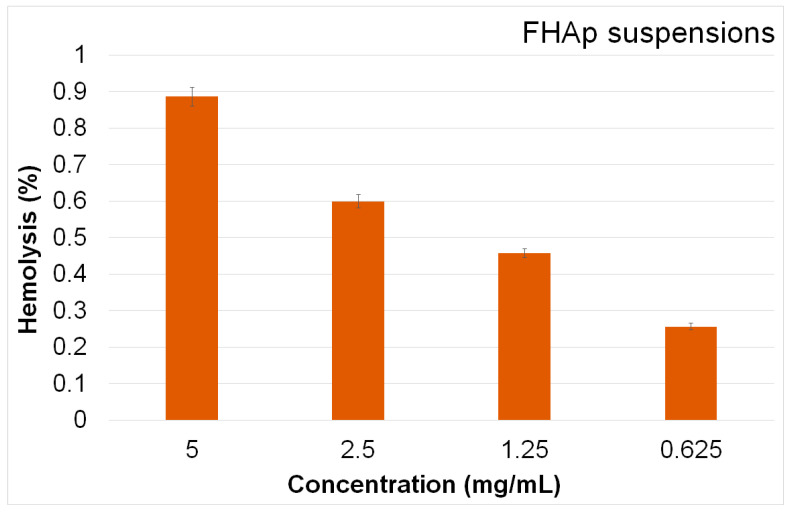
Percentage hemolysis of sheep red blood cells (RBCs) exposed to different concentrations of FHAp suspensions.

The biological properties of the FHAp suspensions were evaluated through both hemocompatibility and biocompatibility studies. The hemolytic properties of the materials suggested their ability to induce lysis or rupture in red blood cells (erythrocytes), therefore indicating the biocompatibility of the tested materials. These studies are crucial because they reveal important information about their safety and biocompatibility regarding their use in contact with blood or biological tissues. Materials that exhibit high hemolytic activity can cause damage to red blood cells, release hemoglobin, and cause adverse physiological responses like inflammation, thrombosis, and organ damage, making them unsuitable for biomedical applications. On the other hand, materials that possess a low hemolytic index are considered suitable for use in biomedical applications because they pose a minimal risk of adverse reactions and have better compatibility with biological systems. The hemocompatibility of the FHAp suspensions was tested according to their hemolytic activity. The results are depicted in Figure 12.

The data obtained through the hemocompatibility assays showed that none of the tested concentrations of FHAp suspensions caused hemolysis. Furthermore, the values obtained were well within the acceptable hemocompatibility limits for a biomaterial. The results highlighted that the FHAp suspensions exhibited a hemolytic activity lower than 1%, with the hemolysis index increasing with concentration. The results of the hemocompatibility assays demonstrated that the FHAp suspensions exhibited a low hemolytic index, making them suitable for further cytotoxicity assessments to confirm their efficacy and safety in biomedical applications [51].

**Figure 13:**
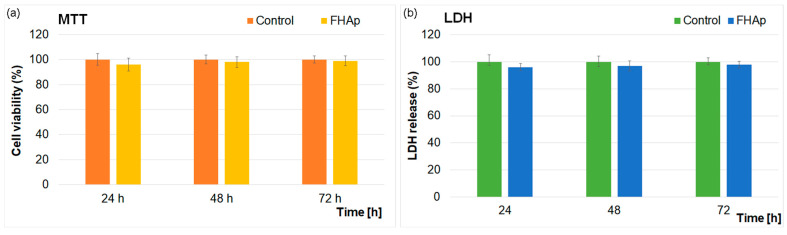
Biocompatibility: (**a**) cell viability (%) determined through MTT assay and (**b**) LDH release (%) of hFOB 1.19 cells exposed to FHAp suspensions at different time intervals; (*p* > 0.05) in all comparisons.

The cytotoxic response of the FHAp suspensions was investigated with the aid of MTT cell viability and LDH release analyses. The cytotoxic response of the FHAp suspensions towards hFOB 1.19 cells was evaluated at three different time intervals (24, 48 and 72 h). The results of the LDH release and MTT cell viability assays are depicted graphically in Figure 13a,b. The results highlighted that the FHAp suspensions did not exhibit any adverse effects on hFOB 1.19 cells at the tested time intervals. The MTT assay results indicated that the cellular viability after 24 h of incubation with the FHAp suspensions was higher than 96% and reached a value of 99% after 72 h of incubation. The results are in good agreement, confirming the biocompatibility of hydroxyapatite and fluoride-doped hydroxyapatite on different cell lines [13,19,52,53,54,55,56,57,58,59,60,61,62,63,64]. These findings were also confirmed by the results obtained from the extracellular LDH release analysis (Figure 13a). The LDH assay measures the lactate dehydrogenase released into the environment from the cytoplasm due to cell membrane damage. The results obtained from the LDH-specific activity assay confirmed the MTT results and highlighted that the FHAp suspensions did not exhibit any cytotoxic activity against the hFOB 1.19 cells. Both the MTT and LDH results indicated that the FHAp suspension exhibited good biocompatibility towards hFOB 1.19 cells, and all the data suggested that the FHAp suspensions could be successfully used in biomedical applications.

In recent years, the emergence of microbial strains with resistance to conventional antibiotics has become a major global health issue. Due to the limited availability of effective antimicrobial agents and therapies for clinical use, there is a growing need for the development of novel antimicrobial agents. Consequently, the development of new materials with enhanced biological properties and efficacy against drug-resistant microbial strains is of significant global interest. Ongoing research and innovation in this field are crucial to addressing the challenges posed by drug-resistant microorganisms to the global health system. In this study, the antimicrobial activity of FHAp suspensions was evaluated. The antimicrobial activity of the FHAp suspensions was tested against some of the most common microbial strains responsible for the appearance of infections, namely *Staphylococcus aureus* 25923 ATCC, *Escherichia coli* 25922 ATCC, and *Candida albicans* 10231 ATCC strains. The antimicrobial assays were performed at three different time intervals and the experiments were performed in triplicate. The results were depicted graphically as mean ± SD.

**Figure 14:**
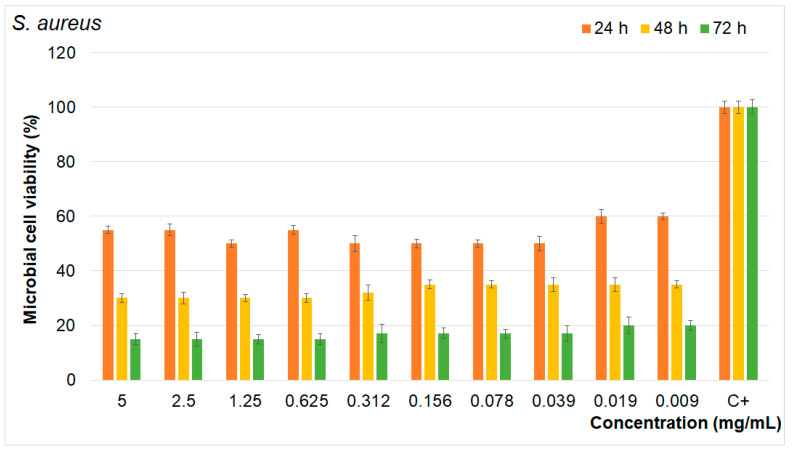
Antimicrobial activity of FHAp suspensions on *S. aureus* at different time intervals.

**Figure 15:**
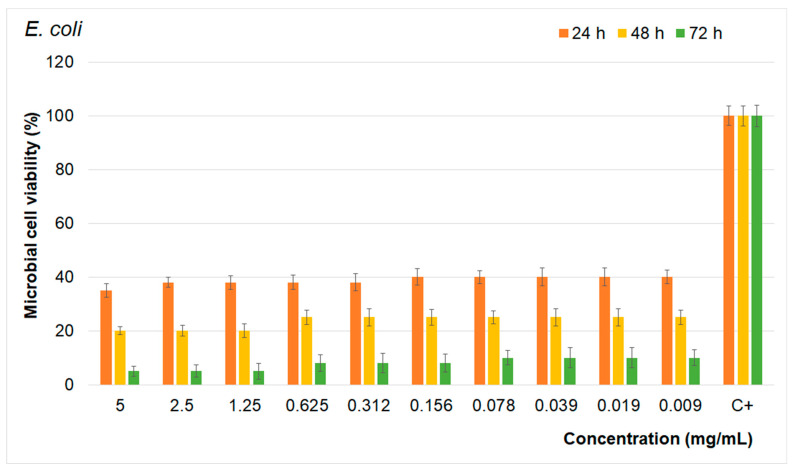
Antimicrobial activity of FHAp suspensions on *E. coli* at different time intervals.

**Figure 16:**
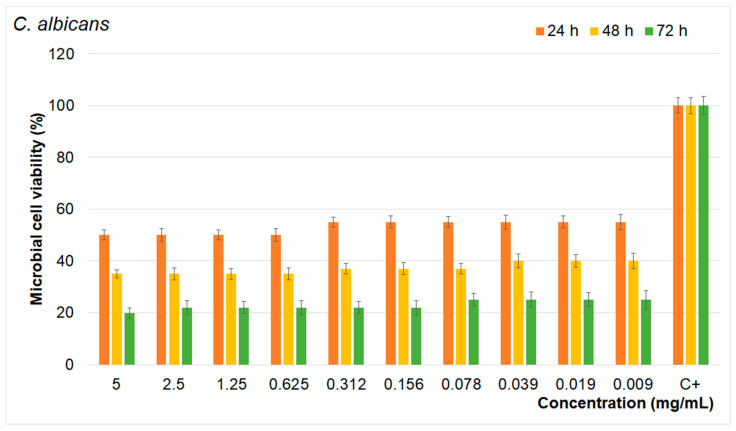
Antimicrobial activity of FHAp suspensions on *C. albicans* at different time intervals.

The results of the quantitative antimicrobial assays presented in Figure 14, Figure 15 and Figure 16 show that the FHAp suspensions inhibited the growth of all the microbial strains tested for all the tested intervals. Moreover, the results emphasize that the FHAp suspensions reduced the microbial cell viability by more than 50% for all the tested microbial strains, even in the first stages of development (first 24 h). On the other hand, the results highlight that the antimicrobial activity of the FHAp suspensions was correlated with the suspension concentrations, as well as with the incubation time and the type of microbial strain that they were tested against.

The results showed that the tested microbial strain was more susceptible to higher concentrations of FHAp suspensions and also that the reduction in microbial cell viability decreased with the increase in the exposure time. Therefore, the most prominent reduction in the microbial cell viability was achieved after 72 h of exposure to the FHAp suspensions in the case of all the tested microbial strains. More than that, the results showed that the microbial strains most susceptible to the FHAp suspensions was the *E. coli* bacterial strain. As is depicted in Figure 14, Figure 15 and Figure 16, all the tested microbial strains were inhibited by the FHAp suspensions at even lower concentrations of 0.09 mg/mL. The data also suggest that the lowest antimicrobial activity was against the fungal strain *C. albicans*. These results are in good agreement with other studies that have suggested that *C. albicans* appears more resistant to silver fluorapatite than the bacterial species tested [65]. This behavior is attributed to the fact that *Candida albicans* cells are typically surrounded by an exopolymeric substance that protects them from adverse environmental conditions. The results of the antibacterial assays are in good agreement with previous research on the antibacterial properties of fluoride and fluoride composites [13,19,20,53,54,55,56,65,66,67,68,69,70,71,72,73,74]. In their work, Kus-Liskiewicz et al. [65] showed that there is a time-dependent bactericidal effect on the gram-negative *E. coli* strain when it is exposed to pure fluorapatite (FAP). Similar results were obtained by Bala et al. [66] in their studies emphasizing the strong antibacterial activity that is exhibited by calcium fluoride nanoparticles against *Escherichia coli*, *Pseudomonas aeruginosa*, *Bacillus badius*, and *Staphylococcus aureus*. The antimicrobial activity of the FHAp was attributed to the presence of F ions in the HAp lattice. Even though the exact mechanisms responsible for the antimicrobial activity of nanomaterials are still unclear, there are several proposed mechanisms that could be responsible for the antimicrobial activity of fluoride ions. The fluoride ions released from FHA can affect the microbial metabolism in numerous ways. For example, they can inhibit the glycolytic enzyme enolase. More than that, the fluoride complexes can mimic phosphate, forming complexes with ADP at enzyme reaction centers, leading to the inhibition of proton-translocating F-ATPases. ATPase is crucial for maintaining the intracellular pH by pumping out protons, and its inhibition disrupts the microbial metabolism and aciduric capabilities. Beyond that, fluoride ions could inhibit acid-producing bacteria such as *Fusobacterium nucleatum* (F. n) and *Treponema* species, which are periodontal pathogens, thus helping to maintain the acid–base balance necessary for proper osteogenesis.

**Figure 17:**
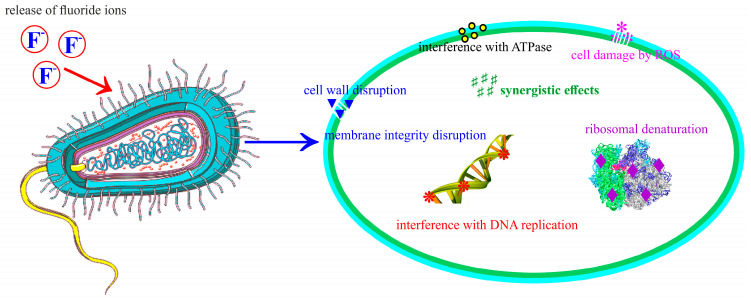
Schematic representation of the antimicrobial mechanism of FHAp suspensions.

A schematic representation of the proposed antimicrobial mechanism of FHAp is depicted in Figure 17.

Fluoride ions are released as bacterial metabolism begins and the pH decreases, which inhibits bacterial growth, stabilizes the microenvironment, and resists the inflammatory process. Another mechanism by which F^−^ ions may interact with bacterial cells is through interference with key metabolic enzymes. However, when developing new fluoride composites, it is essential to consider that the fluoride concentrations required for antimicrobial effects often exceed those needed to reduce apatite solubility [19,20,54,55,56,65,67,68,69,70,71,72,73,74].

The antimicrobial activity of FHAp suspensions could be attributed to a series of mechanisms. First, it can be attributed to the release of fluoride ions that have the ability to disrupt the bacterial metabolism and impair enzyme function, thus compromising the membrane’s integrity and inducing oxidative stress. All of these combined effects lead to the death of bacterial cells, making FHAp suspensions suitable for use in antimicrobial applications.

The results of our studies are in alignment with previously reported studies regarding the antimicrobial activity of fluoride-based materials. Furthermore, our data suggest that FHAp suspensions could be successfully used for the future development of antimicrobial agents.

## 4. Conclusions

In this study, we report the development of a FHAp suspension through an adapted coprecipitation method. The degree of stability of the FHAp suspension was evaluated by zeta potential and ultrasound measurements. Both measurements showed good stability. X-ray diffraction analysis of the FHAp powders resulted in diffraction patterns that resembled pure HAp with a hexagonal structure. The XPS analysis confirms the presence of Ca, P, O, and F in FHAp, and the specific peaks provide valuable insights into the chemical composition and incorporation of fluoride ions. The nanometric dimension of the particles and their tendency to form an agglomerate were revealed by the SEM results. The presence of hydroxyapatite and the lack of supplementary phases in the analyzed samples were underlined by the FTIR results. The results of the in vitro biological assay suggest that FHAp suspensions could be employed for the future development of new antimicrobial agents.

## Data Availability

The original contributions presented in the study are included in the article, further inquiries can be directed to the corresponding authors.
